# Sense of Coherence Mediates the Links between Job Status Prior to Birth and Postpartum Depression: A Structured Equation Modeling Approach

**DOI:** 10.3390/ijerph17176189

**Published:** 2020-08-26

**Authors:** Ganit Goren, Orly Sarid, Philippos Philippou, Alyx Taylor

**Affiliations:** 1 The Spitzer Department of Social Work, Ben Gurion University of the Negev, Beer Sheva 84105, Israel; ganit.goren@012.net.il (G.G.); orlysa@bgu.ac.il (O.S.); philippou.g.philippos@gmail.com (P.P.); 2 School of Psychology, Sport and Physical Activity, AECC University College, Bournemouth BH5 2DF, UK

**Keywords:** birth, employment, job status, postnatal depression, postpartum depression, pregnancy, primiparous, sense of coherence, socio-demographic status, structured equation model

## Abstract

Postpartum depression (PPD) has detrimental effects on the health of the mother, child and family. Socio-demographic variables can influence PPD. Sense of coherence (SOC) is a personal resource that mitigates the experience of stressful events. We hypothesized that SOC would have a protective effect against PPD over time. The aim was to investigate the effects of socio-demographic factors and SOC on PPD at birth (T1) and nine months postpartum (T2). A longitudinal study of primiparous women (*n* = 114; age range 18–47 years) measured PPD, SOC and socio-demographics at T1 and T2. The majority were married, had no economic difficulties and were employed before birth. Results showed that PPD at T1 (15.8%) declined to 6.2% (T2). Job status was positively associated with SOC at T1. The structured equation model accounted for 27% of the variance in PPD (T2). In the first pathway, job status was linked to PPD (T2) via SOC at T1 and T2. In the second, SOC and PPD (T1) and SOC (T2) mediated the link between job status and PPD (T2). Results and clinical implications are discussed in the context of the theory of conservation of resources. An intervention for enhancing SOC is recommended for woman at risk of PPD.

## 1. Introduction

The prevalence of postpartum depression (PPD) in developed countries is between 8 and 20 percent [1,2,3,4,5] including Israel [6].

Postpartum depression affects the woman and her family and can have adverse effects on the cognitive and emotional development of the newly born child [7,8]. The time of onset varies between women [9] and, for many, the symptoms last a relatively short period of three to six months and they recover with no recurrence of symptoms [10]. However, of the 8 to 20 percent of women who experience PPD, a third will report persistent symptoms over one year postpartum [10,11].

Research into risk factors for PPD is ongoing and the range of risk factors already identified suggests that PPD is a bio-psycho-social condition [12]. Identified risk factors include anxiety and depression prior to pregnancy [13,14] or during pregnancy [11,15]. Environmental risk factors that have been identified include the mother experiencing chronic stress [14,16], sexual or physical abuse [17], stressful life events, family conflicts and low level of social support [18,19]. Poor physical health of the mother after birth [16] has also been identified as a risk factor for PPD.

Demographic risk factors include the mother being single [20,21], younger than 20 years [16,22], primiparous [23,24], having low education [16,25] and low economic status [26,27]. In addition, women who did not work prior to giving birth [28,29] or women who do not plan to return to work after the birth have a higher risk of PPD [30].

In contrast to the risk factors, there may be protective factors to consider. There are different personal and social resources which help an individual to maintain wellbeing. There is evidence that social support can be effective for the new mother, including support from her partner, friends, family or colleagues at work [31,32,33,34,35].

Another positive resource is an individual’s ability to perceive and respond to stressful situations, which is known as sense of coherence (SOC) [36,37]. This capacity also demonstrates their confidence in being able to manage the situation through social support and other resources [38]. A person with high SOC believes that they can successfully manage their life events. The theory of SOC forms the conceptual framework of this study. Research groups have examined SOC in relation to coping with medical conditions, finding higher SOC to predict better outcome [39] and better adherence to health behavior change [40].

In relation to childbirth, higher SOC has been found to predict uncomplicated delivery [41] and a lower risk of caesarean section [42]. Another study showed that higher SOC was associated with a lower risk of post-traumatic stress disorder following childbirth [43]. Antenatal cross-sectional studies showed higher SOC to be associated with lower self-rating scale scores for depression [44] and distress [45]. Other studies have revealed an inverse relationship between SOC and PPD [41,46,47]. While Antonovsky did not consider that SOC changes as a result of experiences or over the adult life-span [48], results from studies not associated with childbirth show that SOC may be modified by life experiences [49] and through intervention [50]. What remains unclear is whether SOC changes over the months following birth and what effect SOC has on PPD over the early months after the birth. This is the rationale for the current study.

The aim was to investigate the role of socio-demographic factors and SOC on PPD over time. In consideration of the conceptual framework of SOC, it was hypothesized that job status before the birth would enhance SOC. Secondly, SOC measured immediately after birth and at nine months postpartum would have a protective effect against PPD.

## 2. Materials and Methods

### 2.1. Participants

The study population included 114 women admitted to the maternity wards at the Soroka University Medical Center (Israel). The inclusion criteria were women over 18 years old, who had given birth to their first child and were able to speak and read fluent Hebrew. Using self-reporting questionnaires, data for SOC and symptoms of PPD were collected and evaluated at two time-points: (T1) and (T2). Women with elevated depression scores indicating risk of depressive disorder, those acknowledging thoughts of self-harm and those about whom healthcare professionals had concerns were encouraged and assisted to access appropriate referral pathways. The socio-demographic data (Table 1) showed that the women who took part in the study had a mean age of 29.5 years (SD 4.47; range 21–47), that 86.8% were married or cohabitating, 82.5% were born in Israel, 48.2% held an academic degree, 72.8% reported they had no financial difficulty, and 79.9% of the participants were employed prior to childbirth; among them, 55.3% held full-time jobs. The socio-demographic characteristics of the women who did not complete the second part of the study (*n* = 60) were compared with the characteristics of those who did. There was no significant difference between the two groups for any characteristic.

### 2.2. Procedure

This study was designed as a two-phase prospective longitudinal study. Data were collected between March 2014 and November 2015. In the first phase (T1), women on the ward were screened according to the inclusion criteria and all of those who were eligible were invited to join the study 24–48 h after delivery. Recruitment ceased when the target sample of 200 was reached. Women who agreed to participate in the study were asked to complete self-report questionnaires to measure their sense of coherence and symptoms of depression, which they answered independently. In the second phase (T2), the women who had completed the first phase of the study were contacted by telephone approximately nine months after the birth. Women who agreed to participate in the second phase answered the self-report questionnaires by phone or received a link to answer via the internet. Of the 200 women invited to take part, 70% (*n* = 174) completed the questionnaires at T1, and 65.5% of the women who answered questionnaires at T1 completed questionnaires at T2, providing 114 complete sets of data for analysis. This response rate matches that obtained in similar previous research [47].

### 2.3. Measures

The socio-demographic data collected included age, education, marital status, country of birth, family income status, degree of religiosity and job status. For the purpose of this study, the family income status was used to determine the socioeconomic status and was classified into one of two categories: financial difficulties or no financial difficulties. Financial difficulties were identified as having difficulty to pay for essential needs—for example, for food and electricity. The degree of religiosity was categorized as either secular or religious. The mothers were asked to indicate their job status, which was grouped into three categories: no work, part-time work or full-time work. The full socio-demographic data were taken at T1, while job status and family income status were reported at T2.

The symptoms of PPD were assessed using the Edinburgh Postnatal Depression Scale (EPDS) [51], which has been validated worldwide as an effective screening tool for risk of postpartum depression [52,53]. Examples of items on the EPDS include, “I have been able to laugh and see the funny side of things” and “I have felt sad or miserable”. The Hebrew version has also been validated [54,55]. The scale focuses on the cognitive and affective features of depression and requires the respondent to reflect over the seven days prior to completing it. Each item in the ten-item self-report instrument is scored on a four-point Likert scale according to the severity of the symptoms. The minimum and maximum total scores are 0 and 30, respectively. The EPDS scores are divided into three levels: a score between 10 to 12 indicates mild PPD while a score of 13 and above indicates a major PPD. A positive score on question number 10 asking for thoughts of self-harm is considered major PPD, regardless of the total score [52,56]. The Cronbach’s α coefficient of the EPDS was α = 0.78 at T1 and α = 0.80 at T2, indicating good internal consistency.

The SOC was measured using the SOC-13 questionnaire [48,57]. The SOC-13 has been validated worldwide in different languages including English [58], translated and validated in Hebrew by Bernstein and Carmel [59]. Participants rated each statement on a seven-point Likert scale. Examples of items on the SOC-13 include, “Do you have very mixed-up feelings and ideas?” and “How often do you have the feeling that there’s little meaning in the things you do in your daily life?” Answers were summed and ranged between 13 (low SOC) and 91 (high SOC), indicating better coping capacity. The Cronbach’s α coefficient of the SOC was α = 0.74 at T1 and α = 0.71 at T2, indicating good internal consistency.

### 2.4. Data Analysis

Statistical analyses were performed with IBM SPSS Statistics version 21 with AMOS module. Descriptive analysis of the socio-demographic and the psychological data was carried out: frequencies were calculated for categorical data and means and standard deviations for continuous variables (Table 1). The established EPDS threshold scores of 10 for mild depressive symptoms and 13 and above for severe depressive symptoms [51] were used to group the participants for the summary table (Table 2).

The assumptions for the inferential statistical analysis were tested. The SOC data were normally distributed, but the EPDS data required log transformation to normalize the distribution. One outlier was identified in the SOC T2 data by observation of the descriptive statistics and boxplots of the data. The z-score was greater than 3.29, indicating an extreme outlier, and it was then Winsorized. The possible associations between socio-demographic risk factors and psychological variables were examined.

To examine the possible direct and indirect effects of SOC at T1 on PPD at T1, SOC at T2 and PPD at T2, a structural equation model (SEM) was constructed. The continuous data for the scores for SOC and for PPD were used in the calculations for the SEM. The full sets of data (*n* = 114) were included in the calculation of the SEM, providing a sample larger than the minimum of 100 suggested for the analysis by Awang, Afthanorhan and Asri [60]. There was a non-significant number (0.5%) of randomly distributed missing data points, which were imputed using the mean value for that variable. The socio-demographic variables were entered as predictors for SOC and PPD. An iterative process was used to select the variables that significantly contributed to the model. The model was estimated by the maximum likelihood estimation method. The quality of the fit of the SEM model was assessed by the inferential statistic X^2^ and the ratio of the X^2^ to degrees of freedom (X^2^/df). Two absolute fit indexes, the standardized root mean square residual (SRMR) and the root mean square error of approximation (RMSEA) were used. Other indices used to assess fit included the comparative fit index (CFI), the Tucker–Lewis index (TLI) and the normed fit index (NFI).

### 2.5. Ethical Consideration

The study was approved by the Helsinki Committee Ethical Review Board of Soroka University Medical Center, code number 0006-13SOR. All the women who agreed to take part gave their written informed consent. Women who scored above 10 on the EPDS were referred for support to health professionals in the community. None of the women in the study reported suicidal thoughts on question 10 of the EPDS.

## 3. Results

Results showed that 15.8% of the women reported symptoms indicating PPD at T1 and only 6.2% at T2 (Table 2). The major change in PPD was among the women who reported mild symptoms, which decreased from 13.2% at T1 to only 4.4% at T2. Comparing EPDS scores for the whole study (*n* = 114) showed that PPD at T2 (*M* = 4.01) was significantly lower than T1 (*M* = 4.95), t = −2.36, *p* = 0.02.

The SOC levels at T1 were compared for those with high and low socioeconomic status and there was no significant difference found (File S1). The SOC for women who had a partner (married or cohabiting) was higher than SOC for women with no partner at T1 and T2, but the difference was not significant. Only the job status was found to distinguish the SOC of mothers at T1 and T2. The results are presented in Table 3.

Table 3 shows SOC in the mothers at T1 and T2. There was no difference between SOC for those who worked part-time compared to those who worked full-time, so these groups were combined for further analysis. ANOVA revealed a significant main effect of time on the level of SOC for all the women, such that SOC was higher at T2 than T1, *F* (1, 112) = 19.37, *p* < 0.001. However, the difference was found at T1, with a higher SOC level in women who were employed before birth compared with those who were not employed, t (112) = 2.5, *p* = 0.015. At T2, there was no significant difference in SOC between those who had been employed before the birth and those who had not been employed.

The results of correlational analysis (Table 4) showed that SOC and symptoms of PPD were significantly negatively correlated, with a moderate size (*r* = −0.47, *p* < 0.01) at T1 and at T2 (*r* = −0.52, *p* < 0.01). There were also moderate positive correlations between SOC at T1 and SOC and T2 (*r* = 0.47, *p* < 0.01) and PPD at T1 and PPD at T2 (*r* = 0.337, *p* < 0.01).

### Structural Model

**Figure 1:**
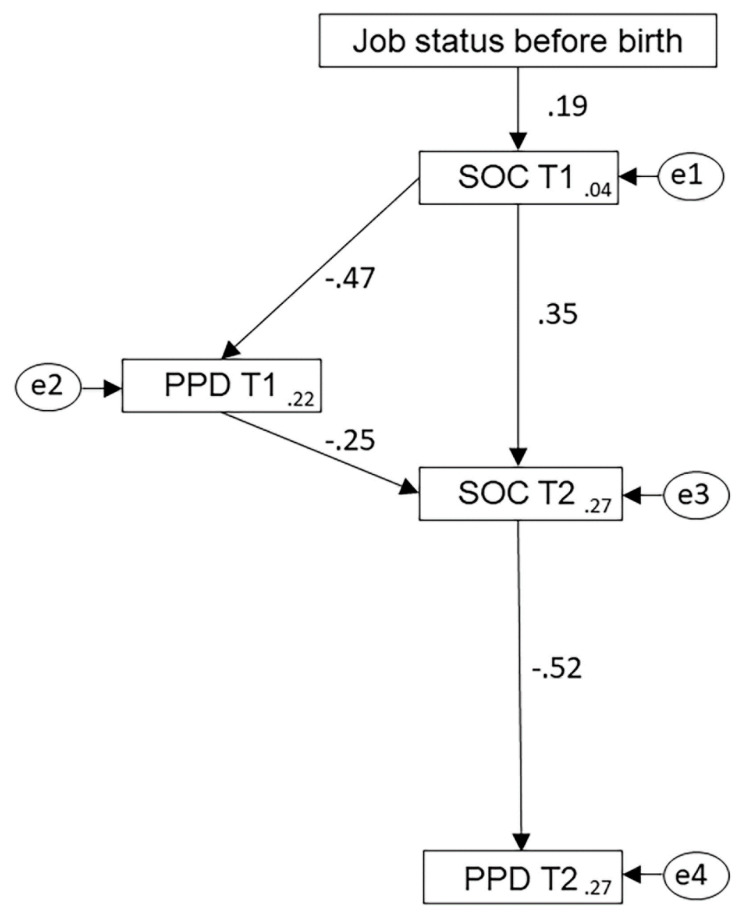
Structural equation model of the relationship between job status prior to the birth, mothers’ sense of coherence (SOC) and postpartum depression (PPD) immediately after the birth (T1) and nine months later (T2). The standardized coefficient for each effect is given beside the corresponding pathway arrow.

The SEM analysis of the predicted model based on data from 114 participants revealed a good fit for the observed data. In the SEM model (Figure 1), standardized coefficients for each effect are given beside the pathway arrow. Inverse relationships are indicated by the negative sign in front of the standardized coefficient. The squared multiple correlation values given in each rectangle show the percentage variance of that variable accounted for by the preceding variable. The AMOS output and SEM model are provided in File S2 and Figure S1.

Table 5 shows the values for the fit, indices including the goodness of fit (GFI = 0.971) and the adjusted goodness of fit (AGFI = 0.914), confirming the model fit. While there were three indices just on the boundary of their indicative range, the majority of the measures reached the indicative range [61].

All the path coefficients were found to be statistically significant at *p* < 0.05. The model accounts for 27% of the variance in PPD at T2. An alternative model was examined, including a direct link between PPD T1 and PPD T2, but the coefficient was not statistically significant (*p* = 0.08), and it did not improve the model fit.

The SEM model of the effect of SOC and early symptoms of PPD on symptoms of PPD at T2 shows two pathways. In the first pathway, work before birth is linked to PPD symptoms at T2 by SOC at T1 and SOC at T2 that served as mediators. Women who worked before birth had relatively higher SOC at T1 (β = 0.19), which led to higher SOC at T2 (β = 0.35). This in turn led to lower symptoms of PPD at T2, with the strongest coefficient of the model (β = −0.52).

In the second pathway, SOC and PPD at T1 and SOC at T2 mediated the link between job status before birth and PPD at T2. In this path, PPD at T1 was an additional mediator in the link between work before birth and PPD at T2. The indirect effect of SOC at T1 and SOC at T2 through the mediator of PPD at T1 had an effect (β = 0.12), generating a total effect of β = 0.47 on SOC at T2.

As there was a significant positive correlation between PPD at T1 and PPD at T2, a modified model was tested with a direct path from PPD at T1 to PPD at T2. The output showed that the coefficient was not statistically significant (β = 0.187, *p* = 0.08). This confirmed that PPD at T1 and PPD at T2 were not linked together directly but were mediated by SOC at T2.

## 4. Discussion

This study was designed to examine the relationships between socio-demographic parameters, SOC and PPD of primiparous mothers, after birth and nine months postpartum. In consideration of the conceptual framework of SOC, it was hypothesized that job status before the birth would enhance SOC. Our results corroborate the hypothesis, showing that the mother’s SOC immediately after birth was influenced by whether or not she was employed prior to giving birth. Women who worked before birth had higher SOC than women who did not work. Our results agree with previous studies that showed that work was associated with lower symptoms of depression [31]. The positive role of work might be explained by Jahoda’s theory of ideal mental health [62], which identified work as a positive influence. While this theory describes employment in relation to overall mental health rather than SOC, it is interesting to note the five factors associated with employment that could contribute positively to SOC. The factors are time-structure, social contacts and shared experiences, social purposes, status and identity and regular activity. Three of these factors—social contacts, social purposes and status and identity—are rewarding, which, in terms of SOC, would make the work worth the effort put in [63]. The other two factors—time-structure and regular activity—would relate to an agreed and predictable schedule of work. Considering this in terms of SOC, it would contribute positively to the individual’s confidence that they are successfully managing an important part of their life [38,63]. Therefore, all the factors could contribute to increasing the individual’s SOC [38]. Furthermore, distinguishing the different factors of employment Jahoda’s theory [62] suggests that work could still have a positive overall effect even when it includes some negative aspects, such as a demanding work schedule with low flexibility on time [64,65].

The concept of engagement [66] may also be considered in relation to job status and SOC. Schaufeli et al. [66] described engagement as a positive, work-related, fulfilling, cognitive-affective state of mind including high energy and mental resilience, sense of meaningfulness, enthusiasm and immersion. Aspects of engagement, including positive affect, work-related fulfilment and a sense of meaningfulness, correspond to components of SOC [48]. Therefore, it might be predicted that only with optimal measures for all aspects of engagement would the individual be satisfied with their work situation and experience an increase in SOC. However, the results from an eight-year, longitudinal study examining response to work situation by white-collar workers showed that individuals with the highest achievements in their own goals were found to be experiencing reduction in some aspects of engagement [67]. At the same time, Mäkikangas et al. [67] showed that there was an increase in stress-related behavior, indicating that the study participants were able to manage their stress sufficiently to achieve their goals. In terms of SOC, this result could be perceived as a worthwhile effort [48]. Among our participants, an important aspect of employment is that it can provide a degree of financial autonomy and sense of empowerment [68]. In turn, this could increase the mothers’ confidence and enable them to manage events in their lives, which would increase their SOC [42,47].

The results from the current study indicate that both part-time and full-time work were associated with lower PPD symptoms. This suggests that the majority of the women in the study found their own work pattern acceptable and sufficiently rewarding. In other words, the outcome was worth the effort, which would increase their SOC. Previous research by Fall, Goulet and Vezina [28] found that women who were working at the time of their prenatal interview had higher socioeconomic status and reported significantly fewer symptoms of depression than women who had stopped working, non-working housewives and students.

Our second hypothesis was that SOC measured immediately after birth and at nine months postpartum would have a protective effect against PPD. Our hypothesis was supported, as the mothers’ SOC changes across the time-points and SOC value increased several months after birth. A possible explanation for this finding may rely on the protective factors that mothers have after birth—for example, social support. While good social support is a protective factor, poor social support is regarded in the literature as a risk factor [69,70]. Social support includes personal contact by family and friends as well as support from health services and charitable organizations. In our study, the women who were married or cohabiting had higher SOC than those who did not have a partner, which agrees with the previous findings. However, there were only 15 women in our sample who did not have a partner. Future studies with a larger sample size are called for before any firm conclusion can be drawn.

Our results support the above findings while introducing SOC as the mediating link between work and PPD. The mother’s SOC exerted a positive influence over the level of PPD symptoms at both time-points. Our SEM model also illustrates the mediating role of SOC between early PPD and PPD at nine months postpartum. The mediating role of SOC on PPD scores might be better understood by considering the context of birth and care of a new child as a significant life event which can cause distress and anxiety for new mothers. The EPDS is an effective screening tool for PPD, but it measures symptoms of depression and anxiety combined [54,71]. Consequently, a high EPDS score may represent a risk of anxiety more than a risk of depression. The conservation of resources theory [72,73] explains that individuals will experience distress when they feel threatened with a loss of something they value (a resource), when they actually experience a loss, or they do not gain a resource after investing existing resource to gain a more valued one. The anticipated or observed loss of resources could be perceived as a reduction in ability to manage challenges, leading to a reduction in the mother’s SOC. At childbirth, there are many resources that the mother may perceive to be threatened—for example, the health of her baby, her own health, fitness, free time, job status or relationships with family and friends. After the baby is born and the mother has had time to adjust to her new status, she could re-evaluate the resources and may experience less stress and anxiety. These changes would be reflected in increased SOC at T2 and reduced EPDS scores at T2. Therefore, the SEM model shows that a new mother who experiences PPD immediately after birth will not necessarily still have PPD nine months later at T2.

### Limitations

Alongside its merits, the current study has several limitations: the first relates to the way in which social support was evaluated including partnership status, religiosity and work. While these objective measures are relevant, the perception of social support is also a subjective experience. Therefore, for future studies, it might also be helpful to include scales measuring mothers’ perceived levels of support. The second limitation might be also viewed as one of the study’s strengths. We recruited new first-time mothers to avoid contamination of previous birth experiences on mothers’ SOC and PPD levels. However, these experiences might be of importance in understanding the development of personal resources and depressive symptoms over time vis a vis labor experiences. We recommend future study to employ a prospective study design and follow a cohort of mothers from their first pregnancy across several years. The third limitation was the small number of women with a low socioeconomic status, which could be addressed by recruiting participants in a location with greater socioeconomic diversity. The fourth limitation relates to the lack of diversity in the ethno-cultural origins of our participants. Other studies showed that ethno-cultural background has a prominent effect on attitudes, norms and personal resources regarding women’s health behavior [74]. Future research is required in order to study women from different ethno-cultural backgrounds within one country or compare women of similar ethno-cultural origin between countries and examine the effect of ethno-culture on their attitudes and norms towards work and how the latter influence their SOC.

## 5. Implications

To enable the most cost-effective support for women in the perinatal period, health services need specific and sensitive screening tools and effective interventions. The positive effect of SOC on PPD shown in these results suggests that a short-term clinical intervention may help the mothers to build up their SOC and consequently reduce the level of PPD. Previously, cognitive behavioral interventions have been shown to increase SOC for specific groups including cardiovascular patients [75] and health workers [76], which indicates that this method could be most effective. With regard to the timing of the intervention, screening and antenatal intervention for women at risk of PPD might enhance SOC by the time of the birth and reduce early PPD. A second phase of the intervention may be offered postpartum for any woman experiencing symptoms as well as those previously identified as being at risk of PPD.

## 6. Conclusions

Our findings indicate that job status prior to birth is an important resource that is related to higher SOC levels. The job status and SOC were discussed in relation to the theory of ideal mental health. This theory identifies five factors able to contribute positively to mental health including social interaction, status and identity and manageable regular activity. These factors correspond to key aspects of SOC. This suggests that, even when some aspects of employment are not ideal, the overall effect can be positive and increase the SOC.

Higher SOC levels are linked with lower symptoms of PPD. This has been discussed in light of the theory of conservation of resources and supportive literature, which identifies different types of resources which help individuals to maintain their sense of wellbeing and confidence in their ability to cope with demanding or stressful situations. SOC, in this context, is an effective coping resource which helps mothers to manage their first year of motherhood and contributes to lower levels of PPD symptoms at nine months postpartum.

This study has indicated the importance of SOC, which should be considered in addition to socio-demographic risk factors. Other studies have shown that cognitive behavioral intervention enhances SOC levels over time. It is therefore recommended that intervention to teach strategies for enhancing SOC is provided both antenatally and postnatally for women who do not work and are identified through screening to be at risk of PPD.

## Figures and Tables

**Table 1:** Socio-demographic characteristics.

Characteristic	% (*n*)	Mean (SD)	Range
Age		29.51 (4.47)	21–47
Country of origin			
Israel	82.5 (94)		
Other	17.5 (20)		
Language			
Hebrew	85.1 (97)		
Arabic	0.9 (1)		
Russian	13.2 (15)		
Other	0.9 (1)		
Family status			
Married/cohabiting	86.8 (99)		
Alone	13.2 (15)		
Education			
Elementary	0.9 (1)		
Highschool	29.8 (33)		
Above high school	21.9 (25)		
BA	37.7 (43)		
MA or higher	10.5 (12)		
Degree of religiosity			
Secular	39.5 (45)		
Religious	60.5 (69)		
Work before birth			
No	20.2 (23)		
Part-time	24.6 (28)		
Full-time	55.3 (63)		
Socioeconomic status			
Not good (low)	27.2 (31)		
Good (high)	72.8 (83)		
Not stated	1.8 (2)		

**Table 2:** Women grouped by their level of symptoms of postpartum depression at T1 (PPD T1) and T2 (PPD T2).

Group	PPD T1 % (*n*)	PPD T2 % (*n*)
No PPD (EPDS < 10)	84.2 (96)	93.9 (107)
Mild PPD (EPDS 10–12)	13.2 (15)	4.4 (5)
Major PPD (EPDS ≥ 13)	2.6 (3)	1.8 (2)

The Edinburgh Postnatal Depression Scale (EPDS) scores were used to group the data by level of symptoms of depression.

**Table 3:** Sense of coherence level at T1 (SOCT1) and T2 (SOCT2) divided by job status before the birth.

Group	SOC T1			SOC T2	
	N	M	SD	M	SD
Did not work	23	67.98	10.57	74.70	9.53
Part-time	28	73.52	9.42	75.41	10.36
Full-time	63	73.42	9.18	76.24	9.22

Number (N); mean (M); standard deviation (SD).

**Table 4:** Correlation matrix showing unstandardized values of the variables included in the final SEM model.

Variable	1	2	3	4	5
Job status before birth	1				
SOC T1	0.192 *	1			
SOC T2	0.049	0.486 **	1		
PPD T1	−0.214 *	−0.471 **	−0.415 **	1	
PPD T2	0.062	−0.282 **	−0.535 **	0.325 **	1

* *p* < 0.05, ** *p* < 0.01, N = 114.

**Table 5:** Fit indices of the model, with indicative range for each.

Fit Indices	Model Value	Indicative Range ^1^
Likelihood ratio χ^2^	X^2^_(5)_ = 9.356 *p* = 0.096	*p*-value > 0.05
χ^2^/df	1.871	χ^2^/df < 3
Goodness of Fit (GFI)	0.971	GFI ≥ 0.95
Adjusted Goodness of Fit (AGFI)	0.914	AGFI ≥ 0.90
Normed Fit Index (NFI)	0.918	NFI ≥ 0.95
Tucker–Lewis Index (TLI)	0.917	TLI ≥ 0.95
Comparative Fit Index (CFI)	0.958	CFI ≥ 0.90
Root Mean Square Error of Approximation (RMSEA)	0.088	RMSEA < 0.08
Standardized Root Mean Square Residual (SRMR)	0.059	SRMR < 0.08

^1^ Values for the indicative range provided in [61].

## Supplementary Materials

The following are available online at https://www.mdpi.com/1660-4601/17/17/6189/s1, File S1: Demographic data - completed compared with drop-outs; File S2: SPSS AMOS output data for the final structural equation model; Figure S1: Structural equation model drawn with SPSS AMOS.

## References

[1] Hahn-Holbrook J., Cornwell-Hinrichs T., Anaya I. (2018). Economic and health predictors of national postpartum depression prevalence: A systematic review, meta-analysis, and meta-regression of 291 studies from 56 countries. Front. Psychiatry.

[2] Pereira A.T., Marques M., Soares M.J., Maia B.R., Bos S., Valente J., Nogueira V., Roque C., Madeira N., Macedo A. (2014). Profile of depressive symptoms in women in the perinatal and outside the perinatal period: Similar or not?. J. Affect. Disord..

[3] Schmied V., Johnson M., Naidoo N., Austin M.P., Matthey S., Kemp L., Yeo A. (2013). Maternal mental health in Australia and New Zealand: A review of longitudinal studies. Women Birth.

[4] Simhi M., Sarid O., Cwikel J. (2019). Preferences for mental health treatment for post-partum depression among new mothers. Isr. J. Health Policy Res..

[5] Wisner K.L., Sit D.K., McShea M.C., Rizzo D.M., Zoretich R.A., Hughes C.L., Confer A.L. (2013). Onset timing, thoughts of self-harm, and diagnoses in postpartum women with screen-positive depression findings. JAMA Psychiatry.

[6] Glasser S. (2010). Postpartum depression: A chronicle of health policy development. Isr. J. Psychiatry Relat. Sci..

[7] Bauer A., Parsonage M., Knapp M., Iemmi V., Adelaja B. (2014). Costs of Perinatal Mental Health Problems. http://eprints.lse.ac.uk/59885/1/__lse.ac.uk_storage_LIBRARY_Secondary_libfile_shared_repository_Content_Bauer%2C%20M_Bauer_Costs_perinatal_%20mental_2014_Bauer_Costs_perinatal_mental_2014_author.pdf.

[8] Moore Simas T.A., Huang M.Y., Patton C., Reinhart M., Chawla A.J., Clemson C., Eldar-Lissai A. (2019). The humanistic burden of postpartum depression: A systematic literature review. Curr. Med. Res. Opin..

[9] Putnam K.T., Wilcox M., Robertson-Blackmore E., Sharey K., Bergink V., Munk-Olsen T., Meltzer-Brody S. (2017). Clinical phenotypes of perinatal depression and time of symptom onset: Analysis of data from an international consortium. Lancet Psychiatry.

[10] Vliegen N., Casalin S., Luyten P. (2014). The course of postpartum depression: A review of longitudinal studies. Harv. Rev. Psychiatry.

[11] Underwood L., Waldie K., D’Souza S., Peterson E., Morton S. (2016). A review of longitudinal studies on antenatal and postnatal depression. Arch. Women Ment. Health.

[12] Fazlagić A. (2011). Psychological correlates of postpartum depression. Acta Med. Median.

[13] Chojenta C.L., Lucke J.C., Forder P.M., Loxton D.J. (2016). Maternal Health Factors as Risks for Postnatal Depression: A Prospective Longitudinal Study. PLoS ONE.

[14] Yim I.S., Tanner Stapleton L.R., Guardino C.M., Hahn-Holbrook J., Dunkel Schetter C. (2015). Biological and psychosocial predictors of postpartum depression: Systematic review and call for integration. Annu. Rev. Clin. Psychol..

[15] Mccall-Hosenfeld J.S., Phiri K., Schaefer E., Zhu J., Kjerulff K. (2016). Trajectories of depressive symptoms throughout the peri- and postpartum period: Results from the first baby study. J. Women Health.

[16] Shrivastava S.R., Shrivastava P.S., Ramasamy J. (2015). Antenatal and postnatal depression: A public health perspective. J. Neurosci. Rural Pract..

[17] Tebeka S., Strat Y.L., Dubertret C. (2016). Developmental trajectories of pregnant and postpartum depression in an epidemiologic survey. J. Affect. Disord..

[18] Glasser S., Barell V., Boyko V., Ziv A., Lusky A., Shoham A., Hart S. (2000). Postpartum depression in an Israeli cohort: Demographic, psychosocial & medical risk factors. J. Psychosom. Obst. Gyn..

[19] Zhang Y., Jin S. (2016). The impact of social support on postpartum depression: The mediator role of self-efficacy. J. Health Psychol..

[20] Brett K., Barfield W., Williams C. (2008). Prevalence of self-reported postpartum depressive symptoms 17states, 2004–2005. MMWR.

[21] De Baca T.C., Wojcicki J.M., Epel E.S., Adler N.E. (2018). Lack of partner impacts newborn health through maternal depression: A pilot study of low-income immigrant Latina women. Midwifery.

[22] Hymas R., Girard L.C. (2019). Predicting postpartum depression among adolescent mothers: A systematic review of risk. J. Affect. Disorders.

[23] Dørheim S.K., Bondevik G.T., Eberhard-Gran M., Bjorvatn B. (2009). Sleep and depression in postpartum women: A population-based study. Sleep.

[24] Nakano M., Sourander A., Luntamo T., Chudal R., Skokauskas N., Kaneko H. (2020). Early risk factors for postpartum depression: A longitudinal Japanese population-based study. J. Affect. Disord..

[25] Kerstis B., Nohlert E., Öhrvik J., Widarsson M. (2016). Association between depressive symptoms and parental stress among mothers and fathers in early parenthood: A Swedish cohort study. Upsala J. Med. Sci..

[26] Chang F., Lee W., Liu Y., Yang J., Chen S., Cheng K., Lin Y., Ho T., Chiu F., Hsu R. (2016). The relationship between economic conditions and postpartum depression in Taiwan: A nationwide population-based study. J. Affect. Disord..

[27] Goyal D., Gay C., Lee K.A. (2010). How much does low socioeconomic status increase the risk of prenatal and postpartum depressive symptoms in first-time mothers?. Women Health Issues.

[28] Fall A., Goulet L., Vézina M. (2013). Comparative study of major depressive symptoms among pregnant women by employment status. Springerplus.

[29] Katon W., Russo J., Gavin A. (2014). Predictors of postpartum depression. J. Women Health.

[30] Glasser S., Stoski E., Kneler V., Magnezi R. (2011). Postpartum depression among Israeli Bedouin women. Arch. Women Ment. Health.

[31] Gjerdingen D., McGovern P., Attanasio L., Johnson P.J., Kozhimannil K.B. (2014). Maternal depressive symptoms, employment, and social support. J. Am. Board Fam. Med..

[32] Howell E.A., Mora P.A., DiBonaventura M.D., Leventhal H. (2009). Modifiable factors associated with changes in postpartum depressive symptoms. Arch. Women Ment. Health.

[33] Xie R.H., He G., Koszycki D., Walker M., Wen S.W. (2009). Prenatal social support, postnatal social support, and postpartum depression. Ann. Epidemiol..

[34] O’Neill P., Cycon A., Friedman L. (2019). Seeking social support and postpartum depression: A pilot retrospective study of perceived changes. Midwifery.

[35] Bielinski-Blattmann D., Lemola S., Jaussi C., Stadlmayr W., Grob A. (2009). Postpartum depressive symptoms in the first 17 months after childbirth: The impact of an emotionally supportive partnership. Int. J. Public Health.

[36] Antonovsky A. (1979). Health, Stress and Coping: New Perspectives on Mental and Physical Well-Being.

[37] Antonovsky A., Marks D.F. (2002). The Health Psychology Reader.

[38] Eriksson M., Lindström B. (2006). Antonovsky’s sense of coherence scale and the relation with health: A systematic review. J. Epidemiol. Commun. Health.

[39] Sinikallio S., Pakarinen M., Tuomainen I., Airaksinen O., Viinamäki H., Aalto T.J. (2019). Preoperative sense of coherence associated with the 10-year outcomes of lumbar spinal stenosis surgery. J. Health Psychol..

[40] Mutikainen S., Föhr T., Karhunen L., Kolehmainen M., Kainulainen H., Lappalainen R., Kujala U.M. (2015). Predictors of increase in physical activity during a 6-month follow-up period among overweight and physically inactive healthy young adults. J. Exerc. Sci Fit..

[41] Oz Y., Sarid O., Peleg R., Sheiner E. (2009). Sense of coherence predicts uncomplicated delivery: A prospective observational study. J. Psychosom. Obst. Gyn..

[42] Ferguson S., Browne J., Taylor J., Davis D. (2016). Sense of coherence and women׳ s birthing outcomes: A longitudinal survey. Midwifery.

[43] Van Heumen M.A., Hollander M.H., van Pampus M.G., van Dillen J., Stramrood C.A. (2018). Psychosocial predictors of postpartum posttraumatic stress disorder in women with a traumatic childbirth experience. Front. Psychiatry.

[44] Ferguson S., Davis D., Browne J., Taylor J. (2015). Sense of coherence and childbearing choices: A cross sectional survey. Midwifery.

[45] Staneva A., Morawska A., Bogossian F., Wittkowski A. (2016). Pregnancy-specific distress: The role of maternal sense of coherence and antenatal mothering orientations. J. Ment. Health.

[46] Kerstis B., Engström G., Edlund B., Aarts C. (2013). Association between mothers’ and fathers’ depressive symptoms, sense of coherence and perception of their child’s temperament in early parenthood in Sweden. Scand. J. Public Health.

[47] Noyman-Veksler G., Herishanu-Gilutz S., Kofman O., Holchberg G., Shahar G. (2015). Post-natal psychopathology and bonding with the infant among first-time mothers undergoing a caesarian section and vaginal delivery: Sense of coherence and social support as moderators. Psychol. Health.

[48] Antonovsky A., Kalimo R., El-Batawi M., Cooper C.L. (1987). Psychosocial Factors at Work and Their Relation to Health.

[49] Lövheim H., Graneheim U.H., Jonsén E., Strandberg G., Lundman B. (2013). Changes in sense of coherence in old age–a 5-year follow-up of the Umeå 85+ study. Scand. J. Caring Sci..

[50] Berger R., Sarid O., Hurvitz N., Anson O. (2009). Sense of coherence and mood states: Exploring the causal relationships. J. Appl. Soc. Psychol..

[51] Cox J.L., Holden J.M., Sagovsky R. (1987). Detection of postnatal depression: Development of the 10-item Edinburgh Postnatal Depression Scale. Br. J. Psychiatry.

[52] Cox J.L., Holden J. (2003). Perinatal Mental Health: A Guide to the Edinburgh Postnatal Depression Scale.

[53] Horowitz J.A., Goodman J. (2004). A longitudinal study of maternal postpartum depression symptoms. Res. Theor. Nurs. Prac..

[54] Bina R., Harrington D. (2016). The Edinburgh postnatal depression scale: Screening tool for postpartum anxiety as well? Findings from a confirmatory factor analysis of the Hebrew version. Matern Child Health J..

[55] Glasser S., Barell V. (1999). Depression scale for research and identification of postpartum depression. Harefuah.

[56] Gibson J., McKenzie-McHarg K., Shakespeare J., Price J., Gray R. (2009). A systematic review of studies validating the Edinburgh Postnatal Depression Scale in antepartum and postpartum women. Acta Psychiatry Scand..

[57] Antonovsky A. (1993). The structure and properties of the sense of coherence scale. Soc. Sci. Med..

[58] Eriksson M., Lindström B. (2005). Validity of Antonovsky’s sense of coherence scale: A systematic review. J. Epidemiol. Commun. Health.

[59] Bernstein J., Carmel S. (1987). Trait anxiety and the sense of coherence. Psychol. Rep..

[60] Awang Z., Afthanorhan A., Asri M.A.M. (2015). Parametric and non-parametric approach in structural equation modeling (SEM): The application of bootstrapping. Mod. Appl. Sci..

[61] Hooper D., Coughlan J., Mullen M.R. (2008). Structural equation modeling: Guidelines for determining model fit. Electron. J. Bus. Res. Method..

[62] Jahoda M. (1981). Work, employment, and unemployment: Values, theories, and approaches in social research. Am. Psychol..

[63] Liukkonen V., Virtanen P., Vahtera J., Suominen S., Sillanmäki L., Koskenvuo M. (2010). Employment trajectories and changes in sense of coherence. Eur. J. Public Health.

[64] Gilbert-Ouimet M., Trudel X., Brisson C., Milot A., Vézina M. (2014). Adverse effects of psychosocial work factors on blood pressure: Systematic review of studies on demand-control-support and effort-reward imbalance models. Scand. J. Work Environ. Health.

[65] Kivimäki M., Kawachi I. (2015). Work stress as a risk factor for cardiovascular disease. Curr. Cardiol. Rep..

[66] Schaufeli W.B., Salanova M., González-Romá V., Bakker A.B. (2002). The measurement of engagement and burnout: A two sample confirmatory factor analytic approach. J. Happiness Stud..

[67] Mäkikangas A., Hyvönen K., Feldt T. (2017). The energy and identification continua of burnout and work engagement: Developmental profiles over eight years. Burnout Res..

[68] Pratley P. (2016). Associations between quantitative measures of women’s empowerment and access to care and health status for mothers and their children: A systematic review of evidence from the developing world. Soc. Sci. Med..

[69] Bales M., Pambrun E., Melchior M., Glangeaud-Freudenthal N.C., Charles M.A., Verdoux H., Sutter-Dallay A.L. (2015). Prenatal psychological distress and access to mental health care in the ELFE cohort. Eur. Psychiatry.

[70] Leahy-Warren P., Newham J., Alderdice F. (2018). Perinatal social support: Panacea or a pitfall. J. Reprod. Infant Psychol..

[71] Jomeen J., Martin C.R. (2005). Confirmation of an occluded anxiety component within the Edinburgh Postnatal Depression Scale (EPDS) during early pregnancy. J. Reprod. Infant Psychol..

[72] Hobfoll S.E. (1989). Conservation of resources: A new attempt at conceptualizing stress. Am. Psychol..

[73] Hobfoll S.E. (2002). Social and Psychological Resources and Adaptation. Rev. Gen. Psychol..

[74] Sarid O., Shraga Y., Cwikel J., Reuveni H. (2019). Ethno-cultural origins, health beliefs and mothers’ behavior regarding infant vaccinations in Israel. Health Promot. Int..

[75] Malm D., Fridlund B., Ekblad H., Karlström P., Hag E., Pakpour A.H. (2018). Effects of brief mindfulness-based cognitive behavioural therapy on health-related quality of life and sense of coherence in atrial fibrillation patients. Eur. J. Cardiovasc. Nur..

[76] Sarid O., Berger R., Segal-Engelchin D. (2010). The impact of cognitive behavioral interventions on SOC, perceived stress and mood states of nurses. Procedia Soc. Behav. Sci..

