# Supplementary material for: Sense of Coherence Mediates the Links between Job Status Prior to Birth and Postpartum Depression: A Structured Equation Modeling Approach
Source: Int J Environ Res Public Health. 2020 Aug 26;17(17):6189. doi: 10.3390/ijerph17176189 (PMC7504177; doi:10.3390/ijerph17176189)
Supplement: Supplementary file 1 [file ijerph-17-06189-s001.zip › File S2 Model AMOS 6(LGx+10).AmosOutput]

Model AMOS 6(LGx+10).amw


#### C:\Users\User\Desktop\Nayomi Taylor\Data Analysis\Final Model\Model AMOS 6(LGx+10).amw

##### Analysis Summary

##### Date and Time

Date: Thursday, August 13, 2020

Time: 1:54:05 PM

##### Title

Model amos 6(lgx+10): Thursday, August 13, 2020 1:54 PM

##### Groups

##### Group number 1 (Group number 1)

##### Notes for Group (Group number 1)

The model is recursive.

Sample size = 114

##### Variable Summary (Group number 1)

##### Your model contains the following variables (Group number 1)

Observed, endogenous variables

N\_SOC\_T1

N\_SOC\_T2

LG10\_EPDS\_T1and10

work\_CAT

LG10\_EPDS\_T2and10

Unobserved, exogenous variables

e4

e7

e5

e3

e9

##### Variable counts (Group number 1)

|  |  |
| --- | --- |
| Number of variables in your model: | 10 |
| Number of observed variables: | 5 |
| Number of unobserved variables: | 5 |
| Number of exogenous variables: | 5 |
| Number of endogenous variables: | 5 |

##### Parameter Summary (Group number 1)

|  | Weights | Covariances | Variances | Means | Intercepts | Total |
| --- | --- | --- | --- | --- | --- | --- |
| Fixed | 5 | 0 | 0 | 0 | 0 | 5 |
| Labeled | 0 | 0 | 0 | 0 | 0 | 0 |
| Unlabeled | 5 | 0 | 5 | 0 | 0 | 10 |
| Total | 10 | 0 | 5 | 0 | 0 | 15 |

##### Assessment of normality (Group number 1)

| Variable | min | max | skew | c.r. | kurtosis | c.r. |
| --- | --- | --- | --- | --- | --- | --- |
| work\_CAT | .000 | 2.000 | -.711 | -3.101 | -1.058 | -2.307 |
| N\_SOC\_T1 | 50.000 | 91.000 | .060 | .260 | -.723 | -1.577 |
| LG10\_EPDS\_T1and10 | 1.000 | 1.447 | .404 | 1.760 | -.849 | -1.851 |
| N\_SOC\_T2 | 42.000 | 91.000 | -.776 | -3.385 | .670 | 1.460 |
| LG10\_EPDS\_T2and10 | 1.000 | 1.544 | .767 | 3.345 | .747 | 1.627 |
| Multivariate |  |  |  |  | 1.027 | .656 |

##### Observations farthest from the centroid (Mahalanobis distance) (Group number 1)

| Observation number | Mahalanobis d-squared | p1 | p2 |
| --- | --- | --- | --- |
| 49 | 21.194 | .001 | .081 |
| 13 | 17.237 | .004 | .079 |
| 110 | 11.546 | .042 | .857 |
| 112 | 11.444 | .043 | .731 |
| 7 | 10.969 | .052 | .712 |
| 6 | 10.658 | .059 | .664 |
| 2 | 10.629 | .059 | .517 |
| 35 | 10.356 | .066 | .477 |
| 67 | 9.515 | .090 | .710 |
| 3 | 9.260 | .099 | .704 |
| 46 | 8.968 | .110 | .725 |
| 30 | 8.817 | .117 | .689 |
| 29 | 8.810 | .117 | .581 |
| 1 | 8.699 | .122 | .528 |
| 91 | 8.604 | .126 | .470 |
| 9 | 8.277 | .142 | .556 |
| 103 | 8.017 | .155 | .612 |
| 107 | 7.968 | .158 | .541 |
| 82 | 7.802 | .168 | .549 |
| 92 | 7.766 | .170 | .473 |
| 5 | 7.681 | .175 | .433 |
| 74 | 7.576 | .181 | .409 |
| 47 | 7.391 | .193 | .445 |
| 94 | 7.383 | .194 | .360 |
| 104 | 7.139 | .211 | .446 |
| 62 | 6.967 | .223 | .486 |
| 80 | 6.782 | .237 | .541 |
| 12 | 6.759 | .239 | .472 |
| 48 | 6.329 | .276 | .726 |
| 21 | 6.174 | .290 | .764 |
| 25 | 6.132 | .294 | .726 |
| 106 | 6.129 | .294 | .655 |
| 114 | 6.066 | .300 | .630 |
| 19 | 5.997 | .306 | .610 |
| 88 | 5.986 | .308 | .540 |
| 85 | 5.970 | .309 | .474 |
| 75 | 5.798 | .326 | .551 |
| 100 | 5.715 | .335 | .550 |
| 4 | 5.632 | .344 | .549 |
| 51 | 5.381 | .371 | .705 |
| 64 | 5.330 | .377 | .682 |
| 76 | 5.325 | .378 | .614 |
| 24 | 5.143 | .399 | .712 |
| 20 | 5.089 | .405 | .694 |
| 105 | 5.047 | .410 | .665 |
| 14 | 4.899 | .428 | .735 |
| 28 | 4.843 | .435 | .722 |
| 17 | 4.813 | .439 | .684 |
| 83 | 4.782 | .443 | .647 |
| 38 | 4.666 | .458 | .694 |
| 86 | 4.629 | .463 | .664 |
| 10 | 4.484 | .482 | .741 |
| 66 | 4.432 | .489 | .728 |
| 90 | 4.428 | .490 | .667 |
| 109 | 4.293 | .508 | .739 |
| 50 | 4.253 | .514 | .716 |
| 108 | 4.195 | .522 | .712 |
| 93 | 4.137 | .530 | .708 |
| 41 | 4.101 | .535 | .681 |
| 42 | 4.063 | .540 | .654 |
| 68 | 4.040 | .544 | .610 |
| 31 | 3.999 | .550 | .586 |
| 77 | 3.995 | .550 | .517 |
| 23 | 3.983 | .552 | .457 |
| 33 | 3.966 | .554 | .404 |
| 26 | 3.763 | .584 | .583 |
| 59 | 3.753 | .586 | .521 |
| 87 | 3.707 | .592 | .505 |
| 113 | 3.704 | .593 | .433 |
| 8 | 3.682 | .596 | .386 |
| 102 | 3.642 | .602 | .362 |
| 11 | 3.563 | .614 | .388 |
| 73 | 3.485 | .626 | .413 |
| 101 | 3.451 | .631 | .382 |
| 69 | 3.376 | .642 | .404 |
| 16 | 3.358 | .645 | .353 |
| 71 | 3.333 | .649 | .311 |
| 45 | 3.254 | .661 | .338 |
| 99 | 3.178 | .673 | .361 |
| 60 | 3.090 | .686 | .403 |
| 52 | 3.070 | .689 | .352 |
| 32 | 3.060 | .691 | .291 |
| 22 | 2.869 | .720 | .472 |
| 34 | 2.826 | .727 | .452 |
| 79 | 2.706 | .745 | .546 |
| 15 | 2.685 | .748 | .492 |
| 89 | 2.562 | .767 | .590 |
| 96 | 2.519 | .774 | .570 |
| 40 | 2.508 | .775 | .497 |
| 65 | 2.462 | .782 | .479 |
| 111 | 2.434 | .786 | .431 |
| 44 | 2.410 | .790 | .378 |
| 81 | 2.300 | .806 | .454 |
| 98 | 2.249 | .814 | .439 |
| 39 | 2.212 | .819 | .402 |
| 54 | 2.094 | .836 | .492 |
| 36 | 2.040 | .844 | .478 |
| 53 | 2.025 | .846 | .400 |
| 61 | 1.944 | .857 | .425 |
| 97 | 1.914 | .861 | .368 |

##### Models

##### Default model (Default model)

##### Notes for Model (Default model)

##### Computation of degrees of freedom (Default model)

|  |  |
| --- | --- |
| Number of distinct sample moments: | 15 |
| Number of distinct parameters to be estimated: | 10 |
| Degrees of freedom (15 - 10): | 5 |

##### Result (Default model)

Minimum was achieved

Chi-square = 9.356

Degrees of freedom = 5

Probability level = .096

##### Group number 1 (Group number 1 - Default model)

##### Estimates (Group number 1 - Default model)

##### Scalar Estimates (Group number 1 - Default model)

##### Maximum Likelihood Estimates

##### Regression Weights: (Group number 1 - Default model)

|  |  |  | Estimate | S.E. | C.R. | P | Label |
| --- | --- | --- | --- | --- | --- | --- | --- |
| N\_SOC\_T1 | <--- | work\_CAT | 2.331 | 1.122 | 2.078 | .038 |  |
| LG10\_EPDS\_T1and10 | <--- | N\_SOC\_T1 | -.005 | .001 | -5.685 | \*\*\* |  |
| N\_SOC\_T2 | <--- | LG10\_EPDS\_T1and10 | -21.452 | 7.663 | -2.800 | .005 |  |
| N\_SOC\_T2 | <--- | N\_SOC\_T1 | .347 | .089 | 3.895 | \*\*\* |  |
| LG10\_EPDS\_T2and10 | <--- | N\_SOC\_T2 | -.006 | .001 | -6.542 | \*\*\* |  |

##### Standardized Regression Weights: (Group number 1 - Default model)

|  |  |  | Estimate |
| --- | --- | --- | --- |
| N\_SOC\_T1 | <--- | work\_CAT | .192 |
| LG10\_EPDS\_T1and10 | <--- | N\_SOC\_T1 | -.472 |
| N\_SOC\_T2 | <--- | LG10\_EPDS\_T1and10 | -.254 |
| N\_SOC\_T2 | <--- | N\_SOC\_T1 | .354 |
| LG10\_EPDS\_T2and10 | <--- | N\_SOC\_T2 | -.524 |

##### Variances: (Group number 1 - Default model)

|  |  |  | Estimate | S.E. | C.R. | P | Label |
| --- | --- | --- | --- | --- | --- | --- | --- |
| e3 |  |  | .631 | .084 | 7.517 | \*\*\* |  |
| e4 |  |  | 89.738 | 11.939 | 7.517 | \*\*\* |  |
| e5 |  |  | .010 | .001 | 7.517 | \*\*\* |  |
| e7 |  |  | 65.019 | 8.650 | 7.517 | \*\*\* |  |
| e9 |  |  | .008 | .001 | 7.517 | \*\*\* |  |

##### Squared Multiple Correlations: (Group number 1 - Default model)

|  |  |  | Estimate |
| --- | --- | --- | --- |
| work\_CAT |  |  | .000 |
| N\_SOC\_T1 |  |  | .037 |
| LG10\_EPDS\_T1and10 |  |  | .222 |
| N\_SOC\_T2 |  |  | .275 |
| LG10\_EPDS\_T2and10 |  |  | .275 |

##### Matrices (Group number 1 - Default model)

##### Total Effects (Group number 1 - Default model)

|  | work\_CAT | N\_SOC\_T1 | LG10\_EPDS\_T1and10 | N\_SOC\_T2 |
| --- | --- | --- | --- | --- |
| N\_SOC\_T1 | 2.331 | .000 | .000 | .000 |
| LG10\_EPDS\_T1and10 | -.013 | -.005 | .000 | .000 |
| N\_SOC\_T2 | 1.083 | .465 | -21.452 | .000 |
| LG10\_EPDS\_T2and10 | -.006 | -.003 | .126 | -.006 |

##### Standardized Total Effects (Group number 1 - Default model)

|  | work\_CAT | N\_SOC\_T1 | LG10\_EPDS\_T1and10 | N\_SOC\_T2 |
| --- | --- | --- | --- | --- |
| N\_SOC\_T1 | .192 | .000 | .000 | .000 |
| LG10\_EPDS\_T1and10 | -.090 | -.472 | .000 | .000 |
| N\_SOC\_T2 | .091 | .474 | -.254 | .000 |
| LG10\_EPDS\_T2and10 | -.048 | -.248 | .133 | -.524 |

##### Direct Effects (Group number 1 - Default model)

|  | work\_CAT | N\_SOC\_T1 | LG10\_EPDS\_T1and10 | N\_SOC\_T2 |
| --- | --- | --- | --- | --- |
| N\_SOC\_T1 | 2.331 | .000 | .000 | .000 |
| LG10\_EPDS\_T1and10 | .000 | -.005 | .000 | .000 |
| N\_SOC\_T2 | .000 | .347 | -21.452 | .000 |
| LG10\_EPDS\_T2and10 | .000 | .000 | .000 | -.006 |

##### Standardized Direct Effects (Group number 1 - Default model)

|  | work\_CAT | N\_SOC\_T1 | LG10\_EPDS\_T1and10 | N\_SOC\_T2 |
| --- | --- | --- | --- | --- |
| N\_SOC\_T1 | .192 | .000 | .000 | .000 |
| LG10\_EPDS\_T1and10 | .000 | -.472 | .000 | .000 |
| N\_SOC\_T2 | .000 | .354 | -.254 | .000 |
| LG10\_EPDS\_T2and10 | .000 | .000 | .000 | -.524 |

##### Indirect Effects (Group number 1 - Default model)

|  | work\_CAT | N\_SOC\_T1 | LG10\_EPDS\_T1and10 | N\_SOC\_T2 |
| --- | --- | --- | --- | --- |
| N\_SOC\_T1 | .000 | .000 | .000 | .000 |
| LG10\_EPDS\_T1and10 | -.013 | .000 | .000 | .000 |
| N\_SOC\_T2 | 1.083 | .118 | .000 | .000 |
| LG10\_EPDS\_T2and10 | -.006 | -.003 | .126 | .000 |

##### Standardized Indirect Effects (Group number 1 - Default model)

|  | work\_CAT | N\_SOC\_T1 | LG10\_EPDS\_T1and10 | N\_SOC\_T2 |
| --- | --- | --- | --- | --- |
| N\_SOC\_T1 | .000 | .000 | .000 | .000 |
| LG10\_EPDS\_T1and10 | -.090 | .000 | .000 | .000 |
| N\_SOC\_T2 | .091 | .120 | .000 | .000 |
| LG10\_EPDS\_T2and10 | -.048 | -.248 | .133 | .000 |

##### Modification Indices (Group number 1 - Default model)

##### Covariances: (Group number 1 - Default model)

|  |  |  | M.I. | Par Change |
| --- | --- | --- | --- | --- |

##### Variances: (Group number 1 - Default model)

|  |  |  | M.I. | Par Change |
| --- | --- | --- | --- | --- |

##### Regression Weights: (Group number 1 - Default model)

|  |  |  | M.I. | Par Change |
| --- | --- | --- | --- | --- |

##### Bootstrap (Group number 1 - Default model)

##### Bootstrap standard errors (Group number 1 - Default model)

##### Scalar Estimates (Group number 1 - Default model)

##### Regression Weights: (Group number 1 - Default model)

| Parameter | | | SE | SE-SE | Mean | Bias | SE-Bias |
| --- | --- | --- | --- | --- | --- | --- | --- |
| N\_SOC\_T1 | <--- | work\_CAT | 1.132 | .057 | 2.295 | -.036 | .080 |
| LG10\_EPDS\_T1and10 | <--- | N\_SOC\_T1 | .001 | .000 | -.005 | .000 | .000 |
| N\_SOC\_T2 | <--- | LG10\_EPDS\_T1and10 | 8.183 | .409 | -21.190 | .262 | .579 |
| N\_SOC\_T2 | <--- | N\_SOC\_T1 | .084 | .004 | .350 | .003 | .006 |
| LG10\_EPDS\_T2and10 | <--- | N\_SOC\_T2 | .001 | .000 | -.006 | .000 | .000 |

##### Standardized Regression Weights: (Group number 1 - Default model)

| Parameter | | | SE | SE-SE | Mean | Bias | SE-Bias |
| --- | --- | --- | --- | --- | --- | --- | --- |
| N\_SOC\_T1 | <--- | work\_CAT | .092 | .005 | .188 | -.004 | .006 |
| LG10\_EPDS\_T1and10 | <--- | N\_SOC\_T1 | .088 | .004 | -.471 | .000 | .006 |
| N\_SOC\_T2 | <--- | LG10\_EPDS\_T1and10 | .093 | .005 | -.250 | .004 | .007 |
| N\_SOC\_T2 | <--- | N\_SOC\_T1 | .093 | .005 | .361 | .007 | .007 |
| LG10\_EPDS\_T2and10 | <--- | N\_SOC\_T2 | .084 | .004 | -.515 | .009 | .006 |

##### Variances: (Group number 1 - Default model)

| Parameter | | | SE | SE-SE | Mean | Bias | SE-Bias |
| --- | --- | --- | --- | --- | --- | --- | --- |
| e3 |  |  | .058 | .003 | .620 | -.011 | .004 |
| e4 |  |  | 9.447 | .472 | 87.901 | -1.837 | .668 |
| e5 |  |  | .001 | .000 | .010 | .000 | .000 |
| e7 |  |  | 11.329 | .566 | 62.219 | -2.801 | .801 |
| e9 |  |  | .001 | .000 | .008 | .000 | .000 |

##### Squared Multiple Correlations: (Group number 1 - Default model)

| Parameter | | | SE | SE-SE | Mean | Bias | SE-Bias |
| --- | --- | --- | --- | --- | --- | --- | --- |
| work\_CAT |  |  | .000 | .000 | .000 | .000 | .000 |
| N\_SOC\_T1 |  |  | .038 | .002 | .044 | .007 | .003 |
| LG10\_EPDS\_T1and10 |  |  | .082 | .004 | .230 | .008 | .006 |
| N\_SOC\_T2 |  |  | .067 | .003 | .289 | .015 | .005 |
| LG10\_EPDS\_T2and10 |  |  | .085 | .004 | .272 | -.002 | .006 |

##### Matrices (Group number 1 - Default model)

##### Total Effects - Standard Errors (Group number 1 - Default model)

|  | work\_CAT | N\_SOC\_T1 | LG10\_EPDS\_T1and10 | N\_SOC\_T2 |
| --- | --- | --- | --- | --- |
| N\_SOC\_T1 | 1.132 | .000 | .000 | .000 |
| LG10\_EPDS\_T1and10 | .007 | .001 | .000 | .000 |
| N\_SOC\_T2 | .523 | .073 | 8.183 | .000 |
| LG10\_EPDS\_T2and10 | .003 | .001 | .057 | .001 |

##### Standardized Total Effects - Standard Errors (Group number 1 - Default model)

|  | work\_CAT | N\_SOC\_T1 | LG10\_EPDS\_T1and10 | N\_SOC\_T2 |
| --- | --- | --- | --- | --- |
| N\_SOC\_T1 | .092 | .000 | .000 | .000 |
| LG10\_EPDS\_T1and10 | .051 | .088 | .000 | .000 |
| N\_SOC\_T2 | .045 | .071 | .093 | .000 |
| LG10\_EPDS\_T2and10 | .024 | .056 | .055 | .084 |

##### Direct Effects - Standard Errors (Group number 1 - Default model)

|  | work\_CAT | N\_SOC\_T1 | LG10\_EPDS\_T1and10 | N\_SOC\_T2 |
| --- | --- | --- | --- | --- |
| N\_SOC\_T1 | 1.132 | .000 | .000 | .000 |
| LG10\_EPDS\_T1and10 | .000 | .001 | .000 | .000 |
| N\_SOC\_T2 | .000 | .084 | 8.183 | .000 |
| LG10\_EPDS\_T2and10 | .000 | .000 | .000 | .001 |

##### Standardized Direct Effects - Standard Errors (Group number 1 - Default model)

|  | work\_CAT | N\_SOC\_T1 | LG10\_EPDS\_T1and10 | N\_SOC\_T2 |
| --- | --- | --- | --- | --- |
| N\_SOC\_T1 | .092 | .000 | .000 | .000 |
| LG10\_EPDS\_T1and10 | .000 | .088 | .000 | .000 |
| N\_SOC\_T2 | .000 | .093 | .093 | .000 |
| LG10\_EPDS\_T2and10 | .000 | .000 | .000 | .084 |

##### Indirect Effects - Standard Errors (Group number 1 - Default model)

|  | work\_CAT | N\_SOC\_T1 | LG10\_EPDS\_T1and10 | N\_SOC\_T2 |
| --- | --- | --- | --- | --- |
| N\_SOC\_T1 | .000 | .000 | .000 | .000 |
| LG10\_EPDS\_T1and10 | .007 | .000 | .000 | .000 |
| N\_SOC\_T2 | .523 | .054 | .000 | .000 |
| LG10\_EPDS\_T2and10 | .003 | .001 | .057 | .000 |

##### Standardized Indirect Effects - Standard Errors (Group number 1 - Default model)

|  | work\_CAT | N\_SOC\_T1 | LG10\_EPDS\_T1and10 | N\_SOC\_T2 |
| --- | --- | --- | --- | --- |
| N\_SOC\_T1 | .000 | .000 | .000 | .000 |
| LG10\_EPDS\_T1and10 | .051 | .000 | .000 | .000 |
| N\_SOC\_T2 | .045 | .053 | .000 | .000 |
| LG10\_EPDS\_T2and10 | .024 | .056 | .055 | .000 |

##### Bootstrap Confidence (Group number 1 - Default model)

##### Bias-corrected percentile method (Group number 1 - Default model)

##### 90% confidence intervals (bias-corrected percentile method)

##### Scalar Estimates (Group number 1 - Default model)

##### Regression Weights: (Group number 1 - Default model)

| Parameter | | | Estimate | Lower | Upper | P |
| --- | --- | --- | --- | --- | --- | --- |
| N\_SOC\_T1 | <--- | work\_CAT | 2.331 | .796 | 4.828 | .034 |
| LG10\_EPDS\_T1and10 | <--- | N\_SOC\_T1 | -.005 | -.007 | -.004 | .007 |
| N\_SOC\_T2 | <--- | LG10\_EPDS\_T1and10 | -21.452 | -32.461 | -3.859 | .047 |
| N\_SOC\_T2 | <--- | N\_SOC\_T1 | .347 | .197 | .490 | .009 |
| LG10\_EPDS\_T2and10 | <--- | N\_SOC\_T2 | -.006 | -.008 | -.004 | .010 |

##### Standardized Regression Weights: (Group number 1 - Default model)

| Parameter | | | Estimate | Lower | Upper | P |
| --- | --- | --- | --- | --- | --- | --- |
| N\_SOC\_T1 | <--- | work\_CAT | .192 | .068 | .387 | .032 |
| LG10\_EPDS\_T1and10 | <--- | N\_SOC\_T1 | -.472 | -.616 | -.321 | .009 |
| N\_SOC\_T2 | <--- | LG10\_EPDS\_T1and10 | -.254 | -.380 | -.052 | .044 |
| N\_SOC\_T2 | <--- | N\_SOC\_T1 | .354 | .210 | .529 | .009 |
| LG10\_EPDS\_T2and10 | <--- | N\_SOC\_T2 | -.524 | -.654 | -.368 | .008 |

##### Variances: (Group number 1 - Default model)

| Parameter | | | Estimate | Lower | Upper | P |
| --- | --- | --- | --- | --- | --- | --- |
| e3 |  |  | .631 | .547 | .746 | .003 |
| e4 |  |  | 89.738 | 75.655 | 107.515 | .003 |
| e5 |  |  | .010 | .008 | .013 | .002 |
| e7 |  |  | 65.019 | 52.141 | 94.119 | .001 |
| e9 |  |  | .008 | .007 | .010 | .002 |

##### Squared Multiple Correlations: (Group number 1 - Default model)

| Parameter | | | Estimate | Lower | Upper | P |
| --- | --- | --- | --- | --- | --- | --- |
| work\_CAT |  |  | .000 | .000 | .000 | ... |
| N\_SOC\_T1 |  |  | .037 | .005 | .150 | .005 |
| LG10\_EPDS\_T1and10 |  |  | .222 | .103 | .379 | .009 |
| N\_SOC\_T2 |  |  | .275 | .152 | .379 | .028 |
| LG10\_EPDS\_T2and10 |  |  | .275 | .136 | .428 | .008 |

##### Matrices (Group number 1 - Default model)

##### Total Effects (Group number 1 - Default model)

##### Total Effects - Lower Bounds (BC) (Group number 1 - Default model)

|  | work\_CAT | N\_SOC\_T1 | LG10\_EPDS\_T1and10 | N\_SOC\_T2 |
| --- | --- | --- | --- | --- |
| N\_SOC\_T1 | .796 | .000 | .000 | .000 |
| LG10\_EPDS\_T1and10 | -.029 | -.007 | .000 | .000 |
| N\_SOC\_T2 | .384 | .340 | -32.461 | .000 |
| LG10\_EPDS\_T2and10 | -.014 | -.004 | .042 | -.008 |

##### Total Effects - Upper Bounds (BC) (Group number 1 - Default model)

|  | work\_CAT | N\_SOC\_T1 | LG10\_EPDS\_T1and10 | N\_SOC\_T2 |
| --- | --- | --- | --- | --- |
| N\_SOC\_T1 | 4.828 | .000 | .000 | .000 |
| LG10\_EPDS\_T1and10 | -.004 | -.004 | .000 | .000 |
| N\_SOC\_T2 | 2.086 | .583 | -3.859 | .000 |
| LG10\_EPDS\_T2and10 | -.002 | -.002 | .226 | -.004 |

##### Total Effects - Two Tailed Significance (BC) (Group number 1 - Default model)

|  | work\_CAT | N\_SOC\_T1 | LG10\_EPDS\_T1and10 | N\_SOC\_T2 |
| --- | --- | --- | --- | --- |
| N\_SOC\_T1 | .034 | ... | ... | ... |
| LG10\_EPDS\_T1and10 | .033 | .007 | ... | ... |
| N\_SOC\_T2 | .031 | .009 | .047 | ... |
| LG10\_EPDS\_T2and10 | .022 | .012 | .024 | .010 |

##### Standardized Total Effects (Group number 1 - Default model)

##### Standardized Total Effects - Lower Bounds (BC) (Group number 1 - Default model)

|  | work\_CAT | N\_SOC\_T1 | LG10\_EPDS\_T1and10 | N\_SOC\_T2 |
| --- | --- | --- | --- | --- |
| N\_SOC\_T1 | .068 | .000 | .000 | .000 |
| LG10\_EPDS\_T1and10 | -.204 | -.616 | .000 | .000 |
| N\_SOC\_T2 | .030 | .353 | -.380 | .000 |
| LG10\_EPDS\_T2and10 | -.097 | -.355 | .042 | -.654 |

##### Standardized Total Effects - Upper Bounds (BC) (Group number 1 - Default model)

|  | work\_CAT | N\_SOC\_T1 | LG10\_EPDS\_T1and10 | N\_SOC\_T2 |
| --- | --- | --- | --- | --- |
| N\_SOC\_T1 | .387 | .000 | .000 | .000 |
| LG10\_EPDS\_T1and10 | -.028 | -.321 | .000 | .000 |
| N\_SOC\_T2 | .174 | .582 | -.052 | .000 |
| LG10\_EPDS\_T2and10 | -.015 | -.157 | .230 | -.368 |

##### Standardized Total Effects - Two Tailed Significance (BC) (Group number 1 - Default model)

|  | work\_CAT | N\_SOC\_T1 | LG10\_EPDS\_T1and10 | N\_SOC\_T2 |
| --- | --- | --- | --- | --- |
| N\_SOC\_T1 | .032 | ... | ... | ... |
| LG10\_EPDS\_T1and10 | .029 | .009 | ... | ... |
| N\_SOC\_T2 | .035 | .020 | .044 | ... |
| LG10\_EPDS\_T2and10 | .031 | .007 | .027 | .008 |

##### Direct Effects (Group number 1 - Default model)

##### Direct Effects - Lower Bounds (BC) (Group number 1 - Default model)

|  | work\_CAT | N\_SOC\_T1 | LG10\_EPDS\_T1and10 | N\_SOC\_T2 |
| --- | --- | --- | --- | --- |
| N\_SOC\_T1 | .796 | .000 | .000 | .000 |
| LG10\_EPDS\_T1and10 | .000 | -.007 | .000 | .000 |
| N\_SOC\_T2 | .000 | .197 | -32.461 | .000 |
| LG10\_EPDS\_T2and10 | .000 | .000 | .000 | -.008 |

##### Direct Effects - Upper Bounds (BC) (Group number 1 - Default model)

|  | work\_CAT | N\_SOC\_T1 | LG10\_EPDS\_T1and10 | N\_SOC\_T2 |
| --- | --- | --- | --- | --- |
| N\_SOC\_T1 | 4.828 | .000 | .000 | .000 |
| LG10\_EPDS\_T1and10 | .000 | -.004 | .000 | .000 |
| N\_SOC\_T2 | .000 | .490 | -3.859 | .000 |
| LG10\_EPDS\_T2and10 | .000 | .000 | .000 | -.004 |

##### Direct Effects - Two Tailed Significance (BC) (Group number 1 - Default model)

|  | work\_CAT | N\_SOC\_T1 | LG10\_EPDS\_T1and10 | N\_SOC\_T2 |
| --- | --- | --- | --- | --- |
| N\_SOC\_T1 | .034 | ... | ... | ... |
| LG10\_EPDS\_T1and10 | ... | .007 | ... | ... |
| N\_SOC\_T2 | ... | .009 | .047 | ... |
| LG10\_EPDS\_T2and10 | ... | ... | ... | .010 |

##### Standardized Direct Effects (Group number 1 - Default model)

##### Standardized Direct Effects - Lower Bounds (BC) (Group number 1 - Default model)

|  | work\_CAT | N\_SOC\_T1 | LG10\_EPDS\_T1and10 | N\_SOC\_T2 |
| --- | --- | --- | --- | --- |
| N\_SOC\_T1 | .068 | .000 | .000 | .000 |
| LG10\_EPDS\_T1and10 | .000 | -.616 | .000 | .000 |
| N\_SOC\_T2 | .000 | .210 | -.380 | .000 |
| LG10\_EPDS\_T2and10 | .000 | .000 | .000 | -.654 |

##### Standardized Direct Effects - Upper Bounds (BC) (Group number 1 - Default model)

|  | work\_CAT | N\_SOC\_T1 | LG10\_EPDS\_T1and10 | N\_SOC\_T2 |
| --- | --- | --- | --- | --- |
| N\_SOC\_T1 | .387 | .000 | .000 | .000 |
| LG10\_EPDS\_T1and10 | .000 | -.321 | .000 | .000 |
| N\_SOC\_T2 | .000 | .529 | -.052 | .000 |
| LG10\_EPDS\_T2and10 | .000 | .000 | .000 | -.368 |

##### Standardized Direct Effects - Two Tailed Significance (BC) (Group number 1 - Default model)

|  | work\_CAT | N\_SOC\_T1 | LG10\_EPDS\_T1and10 | N\_SOC\_T2 |
| --- | --- | --- | --- | --- |
| N\_SOC\_T1 | .032 | ... | ... | ... |
| LG10\_EPDS\_T1and10 | ... | .009 | ... | ... |
| N\_SOC\_T2 | ... | .009 | .044 | ... |
| LG10\_EPDS\_T2and10 | ... | ... | ... | .008 |

##### Indirect Effects (Group number 1 - Default model)

##### Indirect Effects - Lower Bounds (BC) (Group number 1 - Default model)

|  | work\_CAT | N\_SOC\_T1 | LG10\_EPDS\_T1and10 | N\_SOC\_T2 |
| --- | --- | --- | --- | --- |
| N\_SOC\_T1 | .000 | .000 | .000 | .000 |
| LG10\_EPDS\_T1and10 | -.029 | .000 | .000 | .000 |
| N\_SOC\_T2 | .384 | .030 | .000 | .000 |
| LG10\_EPDS\_T2and10 | -.014 | -.004 | .042 | .000 |

##### Indirect Effects - Upper Bounds (BC) (Group number 1 - Default model)

|  | work\_CAT | N\_SOC\_T1 | LG10\_EPDS\_T1and10 | N\_SOC\_T2 |
| --- | --- | --- | --- | --- |
| N\_SOC\_T1 | .000 | .000 | .000 | .000 |
| LG10\_EPDS\_T1and10 | -.004 | .000 | .000 | .000 |
| N\_SOC\_T2 | 2.086 | .203 | .000 | .000 |
| LG10\_EPDS\_T2and10 | -.002 | -.002 | .226 | .000 |

##### Indirect Effects - Two Tailed Significance (BC) (Group number 1 - Default model)

|  | work\_CAT | N\_SOC\_T1 | LG10\_EPDS\_T1and10 | N\_SOC\_T2 |
| --- | --- | --- | --- | --- |
| N\_SOC\_T1 | ... | ... | ... | ... |
| LG10\_EPDS\_T1and10 | .033 | ... | ... | ... |
| N\_SOC\_T2 | .031 | .030 | ... | ... |
| LG10\_EPDS\_T2and10 | .022 | .012 | .024 | ... |

##### Standardized Indirect Effects (Group number 1 - Default model)

##### Standardized Indirect Effects - Lower Bounds (BC) (Group number 1 - Default model)

|  | work\_CAT | N\_SOC\_T1 | LG10\_EPDS\_T1and10 | N\_SOC\_T2 |
| --- | --- | --- | --- | --- |
| N\_SOC\_T1 | .000 | .000 | .000 | .000 |
| LG10\_EPDS\_T1and10 | -.204 | .000 | .000 | .000 |
| N\_SOC\_T2 | .030 | .043 | .000 | .000 |
| LG10\_EPDS\_T2and10 | -.097 | -.355 | .042 | .000 |

##### Standardized Indirect Effects - Upper Bounds (BC) (Group number 1 - Default model)

|  | work\_CAT | N\_SOC\_T1 | LG10\_EPDS\_T1and10 | N\_SOC\_T2 |
| --- | --- | --- | --- | --- |
| N\_SOC\_T1 | .000 | .000 | .000 | .000 |
| LG10\_EPDS\_T1and10 | -.028 | .000 | .000 | .000 |
| N\_SOC\_T2 | .174 | .203 | .000 | .000 |
| LG10\_EPDS\_T2and10 | -.015 | -.157 | .230 | .000 |

##### Standardized Indirect Effects - Two Tailed Significance (BC) (Group number 1 - Default model)

|  | work\_CAT | N\_SOC\_T1 | LG10\_EPDS\_T1and10 | N\_SOC\_T2 |
| --- | --- | --- | --- | --- |
| N\_SOC\_T1 | ... | ... | ... | ... |
| LG10\_EPDS\_T1and10 | .029 | ... | ... | ... |
| N\_SOC\_T2 | .035 | .023 | ... | ... |
| LG10\_EPDS\_T2and10 | .031 | .007 | .027 | ... |

##### Minimization History (Default model)

| Iteration |  | Negative eigenvalues | Condition # | Smallest eigenvalue | Diameter | F | NTries | Ratio |
| --- | --- | --- | --- | --- | --- | --- | --- | --- |
| 0 | e | 0 | 11.408 |  | 9999.000 | 136.176 | 0 | 9999.000 |
| 1 | e | 0 | 5.015 |  | .761 | 42.605 | 3 | .000 |
| 2 | e | 0 | 4.250 |  | .443 | 10.973 | 1 | .974 |
| 3 | e | 0 | 4.195 |  | .098 | 9.396 | 1 | 1.096 |
| 4 | e | 0 | 3.758 |  | .019 | 9.356 | 1 | 1.021 |
| 5 | e | 0 | 4.041 |  | .001 | 9.356 | 1 | 1.001 |

##### Bootstrap (Default model)

##### Summary of Bootstrap Iterations (Default model)

##### (Default model)

| Iterations | Method 0 | Method 1 | Method 2 |
| --- | --- | --- | --- |
| 1 | 0 | 0 | 0 |
| 2 | 0 | 0 | 0 |
| 3 | 0 | 0 | 0 |
| 4 | 0 | 0 | 0 |
| 5 | 0 | 1 | 0 |
| 6 | 0 | 21 | 0 |
| 7 | 0 | 50 | 0 |
| 8 | 0 | 64 | 0 |
| 9 | 0 | 41 | 0 |
| 10 | 0 | 17 | 0 |
| 11 | 0 | 5 | 0 |
| 12 | 0 | 1 | 0 |
| 13 | 0 | 0 | 0 |
| 14 | 0 | 0 | 0 |
| 15 | 0 | 0 | 0 |
| 16 | 0 | 0 | 0 |
| 17 | 0 | 0 | 0 |
| 18 | 0 | 0 | 0 |
| 19 | 0 | 0 | 0 |
| Total | 0 | 200 | 0 |

0 bootstrap samples were unused because of a singular covariance matrix.

0 bootstrap samples were unused because a solution was not found.

200 usable bootstrap samples were obtained.

##### Bootstrap Distributions (Default model)

##### ML discrepancy (implied vs sample) (Default model)

|  |  |  |
| --- | --- | --- |
|  |  | |-------------------- |
|  | .825 | |\* |
|  | 3.422 | |\*\* |
|  | 6.019 | |\*\*\* |
|  | 8.616 | |\*\*\*\*\*\*\* |
|  | 11.213 | |\*\*\*\*\*\*\*\*\*\*\*\* |
|  | 13.809 | |\*\*\*\*\*\*\*\*\*\*\*\*\*\*\* |
|  | 16.406 | |\*\*\*\*\*\* |
| N = 200 | 19.003 | |\*\*\*\*\*\*\* |
| Mean = 15.685 | 21.600 | |\*\*\*\*\*\* |
| S. e. = .485 | 24.197 | |\*\*\* |
|  | 26.794 | |\*\*\*\* |
|  | 29.391 | |\* |
|  | 31.987 | |\* |
|  | 34.584 | |\*\* |
|  | 37.181 | |\* |
|  |  | |-------------------- |

##### ML discrepancy (implied vs pop) (Default model)

|  |  |  |
| --- | --- | --- |
|  |  | |-------------------- |
|  | 13.092 | |\*\* |
|  | 16.576 | |\*\*\*\*\*\* |
|  | 20.061 | |\*\*\*\*\*\*\*\*\*\*\*\*\*\*\* |
|  | 23.546 | |\*\*\*\*\*\*\*\*\*\*\*\*\*\*\* |
|  | 27.030 | |\*\*\*\*\*\*\*\*\*\* |
|  | 30.515 | |\*\*\*\*\*\*\* |
|  | 33.999 | |\*\*\*\*\*\* |
| N = 200 | 37.484 | |\*\*\*\* |
| Mean = 26.485 | 40.968 | |\*\* |
| S. e. = .605 | 44.453 | |\*\* |
|  | 47.938 | |\* |
|  | 51.422 | |\* |
|  | 54.907 | | |
|  | 58.391 | |\* |
|  | 61.876 | |\* |
|  |  | |-------------------- |

##### K-L overoptimism (unstabilized) (Default model)

|  |  |  |
| --- | --- | --- |
|  |  | |-------------------- |
|  | -50.648 | |\* |
|  | -35.806 | |\*\*\* |
|  | -20.965 | |\*\*\*\*\* |
|  | -6.124 | |\*\*\*\*\*\*\*\*\*\*\*\*\* |
|  | 8.718 | |\*\*\*\*\*\*\*\*\*\*\*\*\*\* |
|  | 23.559 | |\*\*\*\*\*\*\*\*\*\*\*\*\*\* |
|  | 38.400 | |\*\*\*\*\*\*\*\*\*\*\*\*\*\* |
| N = 200 | 53.241 | |\*\*\*\*\*\*\*\*\*\*\*\* |
| Mean = 37.817 | 68.083 | |\*\*\*\*\*\*\*\*\* |
| S. e. = 2.880 | 82.924 | |\*\*\*\*\*\*\*\* |
|  | 97.765 | |\*\*\*\*\* |
|  | 112.607 | |\*\*\* |
|  | 127.448 | |\*\*\* |
|  | 142.289 | |\* |
|  | 157.131 | |\* |
|  |  | |-------------------- |

##### K-L overoptimism (stabilized) (Default model)

|  |  |  |
| --- | --- | --- |
|  |  | |-------------------- |
|  | 8.438 | |\*\*\* |
|  | 14.158 | |\*\*\*\*\*\*\* |
|  | 19.879 | |\*\*\*\*\*\*\*\*\*\*\*\*\*\*\*\*\* |
|  | 25.599 | |\*\*\*\*\*\*\*\*\*\*\*\*\*\*\*\*\*\*\* |
|  | 31.320 | |\*\*\*\*\*\*\*\*\*\*\*\*\*\*\*\*\* |
|  | 37.041 | |\*\*\*\*\*\*\*\*\*\*\*\*\*\* |
|  | 42.761 | |\*\*\*\*\*\*\* |
| N = 200 | 48.482 | |\*\*\*\*\*\*\* |
| Mean = 32.738 | 54.202 | |\*\*\*\* |
| S. e. = 1.006 | 59.923 | |\*\*\*\*\* |
|  | 65.643 | |\* |
|  | 71.364 | | |
|  | 77.084 | |\*\* |
|  | 82.805 | | |
|  | 88.525 | |\* |
|  |  | |-------------------- |

##### Model Fit Summary

##### CMIN

| Model | NPAR | CMIN | DF | P | CMIN/DF |
| --- | --- | --- | --- | --- | --- |
| Default model | 10 | 9.356 | 5 | .096 | 1.871 |
| Saturated model | 15 | .000 | 0 |
| Independence model | 5 | 114.614 | 10 | .000 | 11.461 |

##### RMR, GFI

| Model | RMR | GFI | AGFI | PGFI |
| --- | --- | --- | --- | --- |
| Default model | .078 | .971 | .914 | .324 |
| Saturated model | .000 | 1.000 |  |  |
| Independence model | 11.190 | .672 | .507 | .448 |

##### Baseline Comparisons

| Model | NFI Delta1 | RFI rho1 | IFI Delta2 | TLI rho2 | CFI |
| --- | --- | --- | --- | --- | --- |
| Default model | .918 | .837 | .960 | .917 | .958 |
| Saturated model | 1.000 |  | 1.000 |  | 1.000 |
| Independence model | .000 | .000 | .000 | .000 | .000 |

##### Parsimony-Adjusted Measures

| Model | PRATIO | PNFI | PCFI |
| --- | --- | --- | --- |
| Default model | .500 | .459 | .479 |
| Saturated model | .000 | .000 | .000 |
| Independence model | 1.000 | .000 | .000 |

##### NCP

| Model | NCP | LO 90 | HI 90 |
| --- | --- | --- | --- |
| Default model | 4.356 | .000 | 17.099 |
| Saturated model | .000 | .000 | .000 |
| Independence model | 104.614 | 73.823 | 142.861 |

##### FMIN

| Model | FMIN | F0 | LO 90 | HI 90 |
| --- | --- | --- | --- | --- |
| Default model | .083 | .039 | .000 | .151 |
| Saturated model | .000 | .000 | .000 | .000 |
| Independence model | 1.014 | .926 | .653 | 1.264 |

##### RMSEA

| Model | RMSEA | LO 90 | HI 90 | PCLOSE |
| --- | --- | --- | --- | --- |
| Default model | .088 | .000 | .174 | .198 |
| Independence model | .304 | .256 | .356 | .000 |

##### AIC

| Model | AIC | BCC | BIC | CAIC |
| --- | --- | --- | --- | --- |
| Default model | 29.356 | 30.477 | 56.718 | 66.718 |
| Saturated model | 30.000 | 31.682 | 71.043 | 86.043 |
| Independence model | 124.614 | 125.174 | 138.295 | 143.295 |

##### ECVI

| Model | ECVI | LO 90 | HI 90 | MECVI |
| --- | --- | --- | --- | --- |
| Default model | .260 | .221 | .373 | .270 |
| Saturated model | .265 | .265 | .265 | .280 |
| Independence model | 1.103 | .830 | 1.441 | 1.108 |

##### HOELTER

| Model | HOELTER .05 | HOELTER .01 |
| --- | --- | --- |
| Default model | 134 | 183 |
| Independence model | 19 | 23 |

##### Execution time summary

|  |  |
| --- | --- |
| Minimization: | .000 |
| Miscellaneous: | .142 |
| Bootstrap: | .032 |
| Total: | .174 |
