# Supplementary figures and images for: Sense of Coherence Mediates the Links between Job Status Prior to Birth and Postpartum Depression: A Structured Equation Modeling Approach
Source: Int J Environ Res Public Health. 2020 Aug 26;17(17):6189. doi: 10.3390/ijerph17176189 (PMC7504177; doi:10.3390/ijerph17176189)

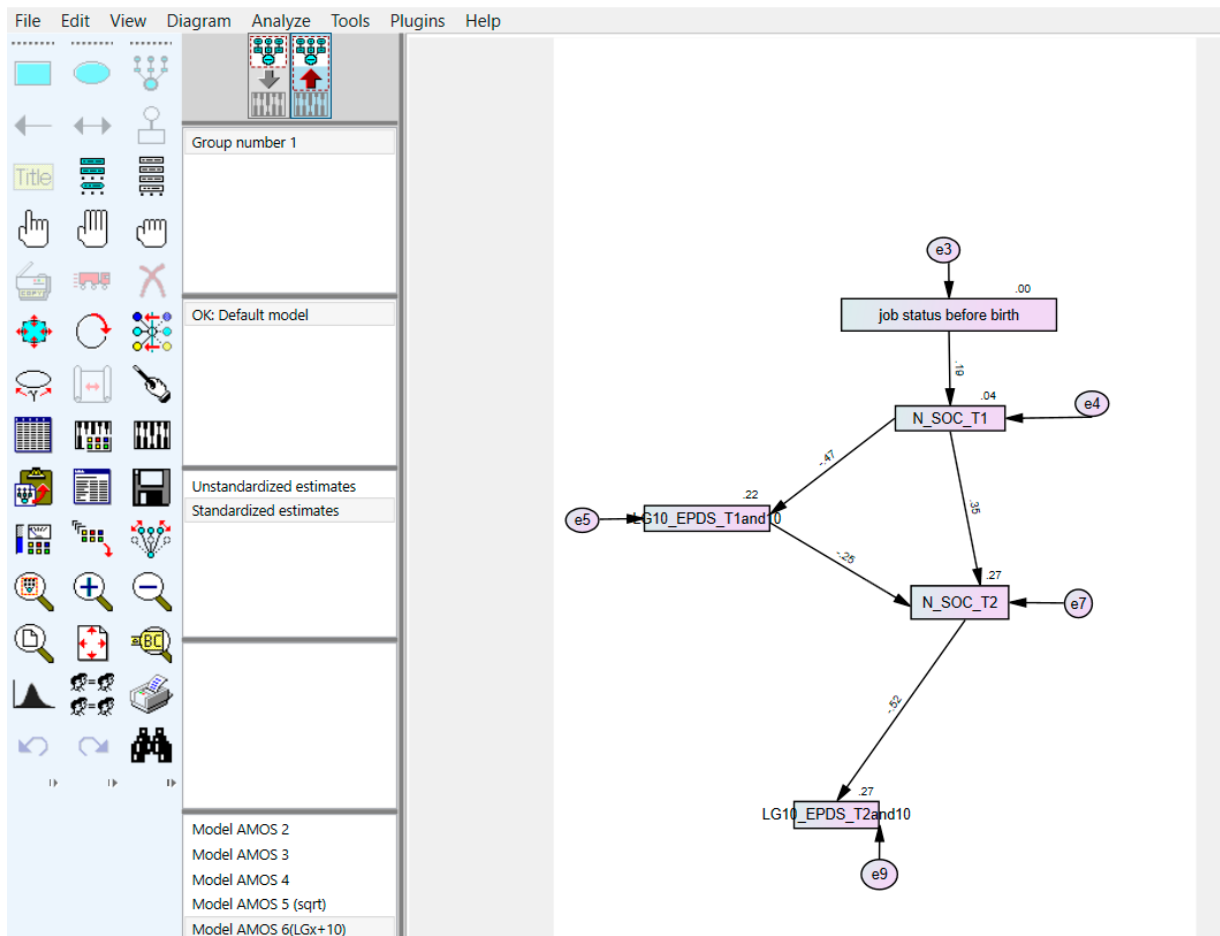

Supplement: Supplementary file 1 [file ijerph-17-06189-s001.zip › ijerph-882355-supplementary.pdf]
